# Academic Literacy Development: What Does It Entail for Multilingual Scholars?

**DOI:** 10.1007/978-3-030-62877-2_1

**Published:** 2021-03-12

**Authors:** Laura-Mihaela Muresan, Concepción Orna-Montesinos

**Affiliations:** 1 Modern Langs & Business Communication, Bucharest University of Economic Studies, Bucharest, Romania; 2 Faculty of Education, University of Zaragoza, Zaragoza, Spain; 3 Bucharest University of Economic Studies, Bucharest, Romania; 4 University of Zaragoza, Zaragoza, Spain

## Abstract

In the introductory chapter, Muresan and Orna-Montesinos provide an overview of the multiple dimensions of academic literacy development, with a focus on its relevance for plurilingual scholars engaged in academic research writing and publishing processes. They situate the ethnographic and pedagogical studies presented in the subsequent chapters within a cognitive/socio-cultural theoretical framework, providing insights into higher education and academic literacy in glocal contexts.

## The Multiple Dimensions of Academic Literacy Development

*Literacy*, as an overarching concept, encompasses multiple dimensions of reading and writing abilities. As a term it has been used to promote a view of writing as social, personal, cognitive and attitudinal practice (Barton, 2007; Barton & Hamilton, 1998; Barton, Hamilton, & Ivanič, 2000; Gee, 1996; Johns, 1997; Lillis & Scott, 2007). In broad terms, development of literacy has been defined and conceptualised as the process of gaining an understanding of disciplinary writing practices and being able to interpret the interconnections between texts and their contexts and the social actions that genres enact (Bazerman & Prior, 2004; Prior, 1998). Literacy studies that have approached the socio-cognitive processes implicated in disciplinary writing activities have underlined the interplay of the individual and the social and, more specifically, the institutional conditions that shape and constrain discourse and rhetorical conventions (Giltrow, 1994; Hyland, 2012; Paré, 2014; Swales, 1990, 2004). Research on literacy as social practice has also stressed the importance of understanding the way in which the social contexts in which the texts are produced constrain the choice of textual features and shape writing practices in various ways (see e.g. Barton et al., 2000; Bazerman & Prior, 2004; Tusting, McCulloch, Bhatt, Hamilton, & Barton, 2019; among others).

Studies of literacy in relation to situated cognition have also brought to the fore another aspect that plays a key role in literacy development, namely, processes of socially situated learning. One compelling argument proposed in this respect is that situated learning takes place through exposure to and peripheral participation in the literate practices of a local disciplinary community (Barton & Hamilton, 1998; Berkenkotter & Huckin, 1995; Johns, 1997). In the scholarly literature, for instance, there is a common enough view that situated literacy development is mainly sustained upon a conscious and intentional awareness of the nature of academic writing, the established norms and conventions, the texts’ intended purposes and the audiences they are meant for. Alongside literacy research, research on LSP (Languages for Specific Purposes) and LAP (Languages for Academic Purposes) further supports the view of learning to write as a process which involves familiarisation with the disciplinary conventions. Wingate (2012) metaphorically refers to it as a “ literacy journey” towards “understand[ing] and control[ling] disciplinary discourses” (p. 26). Interestingly enough, it is further argued that socially situated learning also helps writers become perceptive of intercultural differences across academic cultures, a crucial element in literacy learning (Bardi, 2015; Cumming, 2013; Tardy, 2009).

From the review of the existing research, there also emerges another relevant strand of literature on literacy research that will be taken into consideration in this volume. Essentially, this is a strand that has focused on the individual dimension of writing and brought to the fore the richness and diversity that are inherent to individual experiences in learning how to write academic texts (Bardi, 2015; Corcoran, Englander, & Muresan, 2019). Canagarajah (2020, p. 122) defines literacy development as the process of becoming familiarised with the technical language and the recurring phraseology as well as the genre conventions that shape the production of disciplinary texts. Here, it is also worth recalling Barton’s (2007) contention that literacy is associated with self and identity. In this author’s view, through the writing activity an individual becomes familiarised with and enculturated in the beliefs, values and epistemologies of the disciplinary community that uses the language. Hyland (2012) further explains that identity is intrinsically related to participation in a disciplinary community and “is constructed by individuals appropriating and shaping the discourses which link them to their disciplines” and that “performance of identity” involves individuals situating their contribution to the field “in a particular social world which they reflect and conjure up through the discourses which others anticipate and understand” (p. 1). This view of literacy development has provided new insights into the discourse construction of identity across different types of writing (e.g. expository, persuasive), in the expression of authorial stance and engagement and, more broadly, in writing across different genres—first and foremost, major genres in a research career, such as the PhD dissertation and the research article. Studies on voice and identity have also brought to the fore the interplay of linguistic, cultural and disciplinary norms, and cultural differences at the level of discourse and rhetoric. This range of linguistic, discourse and rhetorical features has been investigated in depth in the literature on English for Research Publication Purposes (ERPP) (Englander & Corcoran, 2019; Flowerdew, 2016) and has given rise to enquiry into processes of self-reflexivity.

Literacy research has also looked into the cognitive and apprenticeship dimensions of literacy in relation to the strategies and resources deployed by novice academics in the writing process, including pre- and post-writing activities. Here, it is worth mentioning that a strand of literature has specifically enquired into aspects of language use, resources and text composing strategies deployed by both novice and senior academics in their scholarly publication endeavours. A key conceptualisation in this terrain has been that of self-regulation, or self-monitoring of one’s writing skills, acquired routines, strategies and resources in the writing process **(**Bazerman, 1981; Beaufort, 1999, 2007; Berkenkotter, Huckin, & Ackerman, 1988; Negretti, 2012; Ramanathan & Atkinson, 1999). Research has further pointed out that previous genre knowledge involves acquired knowledge of content and formal schemata of the genres, as well as the use of conventions, strategies and resources.

As we will also note below, this volume will also seek to illustrate the value of analysing literacy development by delving into the actual processes of teaching/learning how to write disciplinary texts. This will be done critically through thick descriptions of learning to write (Bingham, 2003; Lillis, 2008; Paltridge, Starfield, & Tardy, 2016) and accounts of self-representation, or autoethnographies of literacy development. Studies to date have enquired into the processes experienced by individuals across linguistic and culturally diverse academic settings, either writing in their L1 or in an L2, as described in, for example, Jiang et al. (2010) in China, James (2011) in the Australian context, Kruse (2013) in Europe, and Corcoran et al. (2019) for an international comparison.

Last but not least, research on academic literacy and disciplinary writing is also conclusive regarding the fact that formal instructed learning and pedagogical interventions are supportive of language and literacy development (Wingate, 2012; Wingate & Tribble, 2012 **).** As we expect to illustrate throughout the volume, there is sufficient evidence across geographical contexts worldwide regarding the challenges scholars face in the process of acquiring academic literacy in an L1 and in other languages. Pedagogical interventions can, therefore, play a beneficial role, particularly for enhancing awareness of discourse and genre conventions, and for raising rhetorical consciousness through appropriate tasks and materials (Feak & Swales, 2010; Swales & Feak, 2012).

## Rationale and Aim of the Book

This book looks into the complexity of writing processes, and the multiple dimensions of literacy (socio-historic, socio-cultural, social, cognitive and linguistic) that underlie academic enculturation, the latter conceptualised by Prior and Bilbro (2012) as the processes involving the development of literate practices within an individual’s disciplinary community.^1^ Its broad aim is to illustrate how development of literacy can best be understood if examined from intersecting theoretical perspectives (the language policy dimension, the individual’s cognitive dimension, the socio-literate dimension and the pedagogical dimension). The specific aims of this book are, first, to examine processes of acquisition and mastery of academic literacy in higher education institutions in different geolinguistic regions and, drawing on this international perspective, secondly, to put together empirically-informed explorations of academic literacy development and processes of composing academic texts. The use of academic language(s) and genre repertoires will cover two broad areas, namely, policies and practices, and will specifically look into the implications of both for written production and writing processes, which will be approached from an emic perspective. The main themes discussed in this book relate to the following issues: (1) examination of academic standard guidelines (e.g. norms, expectations, evaluation criteria associated with different academic cultures/systems), (2) academic writing and the development of genre-specific competences in different disciplinary fields, (3) process-orientation, exploring scholarly communities’ (students’ and academics’) views on the writing process and their strategies and challenges related to literacy development at advanced levels/stages. To the best of our knowledge, these different dimensions have not been undertaken and addressed critically in a joint manner. With the contributions reflecting academic endeavours in different geolinguistic contexts, including individual journeys and instructional interventions, we aim to provide research-informed perspectives on how academic writing competences can be acquired, refined, extended, and even transferred from one genre to other genres. The chapters address aspects and conditions of writing across diverse populations that represent different stages in the process of academic enculturation and different levels of expertise in academic writing. With the selected contributions we hope to shed light on issues of academic language(s) use and linguistic repertoires across diverse higher education settings, so as to gain a better understanding of academic writing processes.

Inspired by previous research perspectives on the complex uses of academic languages in changing times (Canagarajah, 2018, 2020; Mauranen, Pérez-Llantada, & Swales, 2020; Palfreyman & van der Walt, 2017; Wu, Mauranen, & Lei, 2020), we would also like to point out that the contributions to this volume will not focus primarily on the challenges faced by scholars who write academic texts in a dominant language (English) that is not their L1. We believe that this has already been discussed very extensively in the scholarly literature. Rather, in this book we seek to zoom into the processes of developing literacy and into accounts of social and situated learning of academic literacy. In doing so, the perspectives and insights offered in this book can make a relevant contribution to the rich diversity of linguacultural backgrounds across academic and research settings, and can be applied across academic writing styles, writing cultures, and rhetorical traditions across languages. In other words, the range of phenomena described in the literature applies to diverse contexts, national systems, cultural traditions, and the claims made will render a more than acceptable level of generalisation. We hope that these decisions serve to foreground the newsworthiness of this volume at a time in which the traditional dichotomy between English-native and non-native English speakers has given way to more timely conceptualisations such as plurilingual competences and multlingualism in academia.

Cumming (2013) sensibly advocates the importance of considering academic literacy development at the intersection of cognitive skills, personal attitudes, social practices and macro-societal structures. In the light of the findings reported in the chapters of this volume, we invite readers to further theoretical and methodological developments in future research to gain deeper insights into the complexities of literacy, disciplinary writing and processes of literacy development in different language contexts.

## Organisation of the Book

The subsequent chapters have been grouped into four thematic sections, even if there are common threads identifiable throughout the book, across sections and geo-linguistic regions. We will see that both novice scholars and mature writers are interested in enhancing their expertise in disciplinary writing and in refining their academic literacies. These endeavours may be constrained by social dynamics and institutional structures or, at times, facilitated by institutional initiatives, or by academic collaboration and networking. It also involves an in-depth understanding of disciplinary discourses and refined skills for constructing a scholarly identity through various linguistic resources. We will also see the positive effects of formal instruction on the scholars’ writing competences across genres and languages, their ability to self-regulate their writing and the perceptions of their writing efficacy.

In exploring the interrelating dimensions of literacy, the various chapters illustrate a range of qualitative research methods employed for describing and interpreting aspects of academic writing processes and academic literacy development. Several of the studies included in this volume draw on mixed-method approaches to enquire into the perceptions yielded from the interviews, while triangulating these data with surveys, questionnaire or textual analysis data of writing samples or writing guidelines. The qualitative and quantitative data are presented in the form of longitudinal studies, text histories or case studies.

### Part I: Language Policies and Academic Literacy

To frame aspects of literacy in institutional ( higher education) contexts, the volume starts with a selection of chapters that provide empirically based reflections on and enquiry into linguistic policies, norms or guidelines regarding the use of both academic English and other academic languages. These contributions also explore the impact of co-existing, controversial academic norms and guidelines on research writing in different linguistic eco-systems.

In Chap. 10.1007/978-3-030-62877-2_2, Hynninen analyses the textual trajectory of a successful research paper, co-authored by six multilingual scholars. Text history data allow her to explore how different actors are allocated literacy broker roles and how they negotiate the writing norms and conventions for their interventions in the process of academic text production. In Chap. 10.1007/978-3-030-62877-2_3, Giannoni offers a critical overview of the language requirements and opportunities for thesis submission in languages other than English at UK universities. The implications of vague linguistic policies for academic literacy development are discussed in the light of recent research on multilingualism in higher education. Chapter 10.1007/978-3-030-62877-2_4 starts with the provocative question, “What constitutes ‘good’ academic writing” for multilingual students in EMI programs? McCambridge critically discusses the interplay of different academic writing norms and underlines the importance of supporting the students’ academic literacy development in ways that would help them to understand and negotiate the meaning of the different kinds of literacy practices and norms they will encounter.

### Part II: Developing and Linking Literacies—Academic, Professional Literacy and Genre Literacy

This section includes studies on academic writing development that draw on ethnographic or ethno-methodological enquiry, with data collected from observation, interviews and questionnaire-based surveys, conducted with graduate, Master and doctoral students, in a variety of disciplines and a diversity of international academic contexts (France, Spain, Iceland, the USA).

Chapter 10.1007/978-3-030-62877-2_5 focuses on the development of literacy in professional and academic contexts using a protocol-assisted modelling (PAM) approach, implemented to help student writers enrolled in a technical communication program to develop audience awareness by constructing a more accurate mental model of how readers think and react to information. Dressen-Hammouda discusses how the PAM method, applied in a French academic setting, contributes to the students’ literacy development by helping them better adapt their attitudes, behaviours and abilities to often unstated and shifting professional expectations. The emergence of situated work-based literacy development is thus linked by the author to mastering professional genres as well as to building disciplinary or professional identity and epistemology.

Chapter 10.1007/978-3-030-62877-2_6 explores the development of academic writing literacy in a group of Spanish doctoral students participating in a writing course. Through a thematic analysis of the difficulties and challenges faced by students in their writing processes in English as an additional language, Guillén-Galve and Vela-Tafalla critically discuss how the acquisition of operational skills appears to be based on the identification of sources and strategies of independent learning and of self-efficacy, complemented by literacy brokering practices.

Chapter 10.1007/978-3-030-62877-2_7 reports on the implementation of an EMI program which seeks to develop students’ academic writing, at the University of Iceland. In order to explore the students’ perceptions, Prinz and Arnbjörnsdóttir have conducted pre- and post-course surveys, with a focus on the students’ ability to regulate academic writing and their writing self-efficiency beliefs. Strategy instruction in writing appeared to help students to transfer conceptual knowledge about academic writing to functional writing skills and, thus, to develop the operational strategies and skills needed to become autonomous writers.

Chapter 10.1007/978-3-030-62877-2_8 builds on an ethnographic study of a group of novice Chemistry researchers (in the US academic context) participating in a series of writing workshops with a focus on science communication genres. McCarty debates the perception of a clear divide between disciplinary writing addressed to specialists and science communication (Sci comm), targeted at non-specialist audiences. These novice writers question the creation of the narrative of science as newsworthy and the incorporation of more nuanced information about the research process. The author concludes that learning to write these new genres to address non-specialist audiences in new contexts imposes a more flexible approach to writing, one in which textual innovation results in the expression of learners’ shifting identity as scientists.

### Part III: Agency, Identity and Self-representation in Literacy Development

The chapters in this section take further the exploration of academic literacy development journeys by placing a special focus on authorial voice, manifestations of identity, or the researcher’s and participants’ self-reflection and self-representation in the process of knowledge co-construction. The truly international dimension of the studies reported on here is reflected not only by the range of academic contexts where the chapter authors are based (Australia, Japan, R. N. Macedonia, Ukraine), but also by the diversity of linguistic and cultural backgrounds of the participants in their research, whose original country contexts included, for instance, Bosnia-and-Herzegovina, Brazil, China, Korea, R. N. Macedonia, Malta, Norway, Portugal, Romania, the UK, Ukraine.

Chapter 10.1007/978-3-030-62877-2_9 focuses on the development of authorial voice in multilingual international students writing their theses in an additional language. Seeking to provide both students and supervisors with clear and pertinent advice on how to develop and express authorial voice, Morton and Storch critically evaluate the treatment of voice in thesis writing guidebooks and online material produced by universities as well as by popular blogs. Their research revealed that the resources targeting students were simplistic, providing only limited advice on authorial voice, and therefore, they stress the role of supervisors in guiding doctoral students to acquire strategies that can contribute to the development of writers’ authorial voice across disciplines.

In Chap. 10.1007/978-3-030-62877-2_10, Bekar and Yakhontova report on a qualitative study of the various aspects of student writers’ *self,* revealed through personal opinions, authoritativeness and presence in the interviews conducted. The study focuses on the manifestation of identity as a linguistic phenomenon, that is, on the discoursal resources which convey the writers’ personal opinions, beliefs, and attitudes, and which are used to justify their actions during the thesis writing process. This scrutiny of linguistic choices sheds light into how the students’ identities and own versions of reality are constructed. It also foregrounds a complex, multiaspectual nature of their self-presentation as *anxious*, *supported*, *independent* or *triumphant* writers.

Chapter 10.1007/978-3-030-62877-2_11 provides an account of the author’s own experience as a facilitator in writing conference groups for graduate students writing their thesis. In her ethnographic study, Mochizuki examines knowledge and meaning co-creation between the participants and herself, as a researcher, aiming to understand the significance of the role of researcher reflexivity in academic literacy development. The findings presented show how her interaction with the participants has contributed to transforming their views and to the negotiation of their identities and goals. The chapter illustrates how direct observation and reflexivity allow the researcher to be actively involved not only in knowledge gathering but also in knowledge construction.

### Part IV: Individual Trajectories in Academic Literacy Development

The studies included here delve into multilingual scholars’ literacy development stages, exploring the challenges they face in constructing academic texts, the strategies they have developed and the resources they are resorting to.

In Chap. 10.1007/978-3-030-62877-2_12, Habibie presents the findings of a case study that explored the strategies, pedagogies and mentorship practices of a multilingual early-career academic in writing for scholarly publication. The author framed his ethnographically oriented study resorting to Tardy’s (2009) notion of genre knowledge, and Lave and Wenger’s (1991) Legitimate Peripheral Participation approach, with the aim of enquiring into the discursive and non- discursive challenges faced by this novice scholar, as well as into the strategies she has developed for enhancing and refining her academic literacies.

In Chap. 10.1007/978-3-030-62877-2_13, Villares explores the perceptions of Spanish multilingual scholars regarding academic literacy development. The main challenges referred to in interviews consisted in both internal factors (personal experiences) and external factors (availability of resources, policy guidelines). In addition, the study reveals the scholars’ perceptions of linguistic disadvantage during the writing and manuscript submission process. The use of both individual learning-by-doing strategies and collaborative strategies highlights the crucial role of the social aspect of academic literacy development. The implementation of policy decisions which foster the enculturation process of novice researchers and the mastery of academic literacy are discussed as a response to insufficient training and institutional support services.

Chapter 10.1007/978-3-030-62877-2_14 explores academics’ perspectives on the development of feedback and scholarly writing literacies. Using concept map-mediated interviews, Gravett provides insights into academics’ experiences on moving from the highly emotive nature of learning from critical feedback and rejection, to taking critical feedback forward in order to offer a considered response. Despite the benefits of the collaborative nature of the peer review process, the study exposes the perception of power inequalities illustrated by the pressures experienced by multilingual scholars writing in English and the concerns about the debilitating and frustrating nature of the review process which might lead to the lack of faith in reviewers’ credibility, motivation or expertise.

Chapter 10.1007/978-3-030-62877-2_15 presents longitudinal interview data from two plurilingual early career researchers participating in a writing for publication tutoring programme. Janssen and Crites explain how the evolution of the participants’ writing practices is connected to the interaction with academic literacy brokers and to the development of their research networks. Their findings show how engagement in networks with different literacy brokers (supervisors, advisors, editors) or collaboration and face-to-face discussion with other members of their community of practice, including friends, colleagues, co-authors, play a crucial role in mediating the publication process.

### Academic Literacy Development After the Spring of 2020: *Quo Vadis*?

In Chap. 10.1007/978-3-030-62877-2_16, Tercero brings to the fore the challenges and changes experienced by academia in the spring of 2020, which caught both students and instructors in Higher Education fairly unprepared. On the basis of the findings of a small-scale exploratory study of instructor and student perceptions of their online writing courses, the author proposes a framework for designing and teaching an online academic writing course for L2 writers. As Tercero concludes, the COVID-19 pandemic might have fostered the expansion of online teaching and learning in the near future in unprecedented ways, which makes the design of quality online teaching and learning a crucial endeavour.

### Power, Identity and Academic Literacy: A Coda

In this *coda* chapter, Englander and Corcoran review recent research on multilingual scholars’ trajectories and experiences in writing academic texts to bring to the fore the need for addressing advanced stages of literacy development from multiple perspectives. After highlighting the conceptual complexity of *literacy*, with its multilayered and intersecting dimensions, the authors engage with several salient themes addressed in the contributions to this volume. They underline the “variety of ethnographic research methodologies” employed in the studies included in this collection, as well as the value of bringing together such a range of “glocalized perspectives from broad geolinguisitic locales”. Englander and Corcoran conclude by problematising possible implications of the current tendencies towards achieving homogeneity in academic publishing and invite for further reflections on the multiple facets of academic knowledge exchange.

## Footnotes

1 Funding support of the Spanish Ministry of Economy and Competitiveness and the European Social Fund (under the project “Ecologies of Genres and An Ecology of Languages: The Dynamics of Local, Cross-border and International Scientific Communication” (project code FFI2015-68638-R MINECO/FEDER) is gratefully acknowledged.
